# Ayahuasca and debut of psychosis, description of a clinical case

**DOI:** 10.1192/j.eurpsy.2025.1620

**Published:** 2025-08-26

**Authors:** E. E. Morales Castellano, P. Rivero Rodriguez, M. Martinez Grimal, J. J. Tascón Cervera, A. M. Morales Rivero

**Affiliations:** 1 Psychiatry, Hospital universitario de gran canaria Dr Negrin, las palmas de gran canaria, Spain

## Abstract

**Introduction:**

The relationship between psychosis and ayahuasca use is a topic of considerable interest and debate in the scientific and medical community. Ayahuasca is traditionally used in shamanic ceremonies in the Amazon, composed mainly of the Banisteriopsis caapi vine and the leaves of Psychotria viridis, which contain DMT (dimethyltryptamine), a powerful hallucinogen.

Ayahuasca induces altered states of consciousness, with experiences that can include visions, deep introspection, and changes in the perception of reality. The experience can be intense and emotionally overwhelming. In individuals with a history of psychosis or psychiatric disorders, ayahuasca may more easily trigger a psychotic episode. The interaction with neurotransmitters and the serotonergic system can influence their appearance.

We report a case of a patient who, after consuming ayahuasca, presented a psychotic episode that required hospitalization. After the initiation of treatment with antipsychotics, the condition had a favorable evolution towards the complete resolution of the symptoms.

Given the increasingly frequent use of ayahuasca in developed societies, the current case highlights the needs to understand, regulate and research the use of ayahuasca. Additionally, raising awareness of the potential risks of ayahuasca use through psychoeducation should be implemented.

**Objectives:**

Describe the case of a psychotic episode induced by ayahuasca: Clinical debut, symptoms and evolution after initiation of treatment.

**Methods:**

Description of a clinical case as well as bibliographic review.

**Results:**

The relationship between hallucinogens and psychosis is a critical area of study due to the intense and potentially destabilizing effects that hallucinogens can have on people with this condition. Hallucinogens can trigger or aggravate psychotic symptoms, which may include hallucinations and delusions. We must keep in mind that hallucinogens can interact negatively with antipsychotics or other medications used to treat psychosis. These interactions may reduce the effectiveness of treatment or cause adverse side effects, as well as affect adherence to treatment, as it may lead to an alteration in the perception of the need for medication or the ability to follow the treatment regimen.

**Conclusions:**

Ayahuasca use can have profound and potentially destabilizing effects, especially in people with a history of psychotic disorders. Although there is therapeutic potential, it is crucial to proceed with caution and under the supervision of trained professionals. Proper preparation and risk assessment are essential to minimize potential adverse effects.

**Disclosure of Interest:**

None Declared

